# Aberrant Methylation of Gene Associated CpG Sites Occurs in Borderline Personality Disorder

**DOI:** 10.1371/journal.pone.0084180

**Published:** 2013-12-19

**Authors:** Stefanie Teschler, Marek Bartkuhn, Natascha Künzel, Christian Schmidt, Steffen Kiehl, Gerhard Dammann, Reinhard Dammann

**Affiliations:** 1 Institute for Genetics, Justus-Liebig-University Giessen, Giessen, Germany; 2 Psychiatric Hospital, Spital Thurgau AG, Münsterlingen, Switzerland; 3 Department of Psychiatry, Basel University Medical School, Basel, Switzerland; The Chinese University of Hong Kong, Hong Kong

## Abstract

Borderline personality disorder (BPD) is a complex psychiatric disease with an increased impact in the last years. While the diagnosis and therapy are well established, little is known on the pathogenesis of borderline personality disorder. Previously, a significant increase in DNA methylation of relevant neuropsychiatric genes in BPD patients has been reported. In our study we performed genome wide methylation analysis and revealed specific CpG sites that exhibited increased methylation in 24 female BPD patients compared to 11 female healthy controls. Bead chip technology and quantitative bisulfite pyrosequencing showed a significantly increased methylation at CpG sites of *APBA2* (1.1 fold) and *APBA3* (1.1 fold), *KCNQ1* (1.5 fold), *MCF2* (1.1 fold) and *NINJ2* (1.2 fold) in BPD patients. For the CpG sites of *GATA4* and *HLCS* an increase in DNA methylation was observed, but was only significant in the bead chip assay. Moreover genome wide methylation levels of blood samples of BPD patients and control samples are similar. In summary, our results show a significant 1.26 fold average increase in methylation at the analyzed gene associated CpG sites in the blood of BPD patients compared to controls samples (p<0.001). This data may provide new insights into epigenetic mechanisms underlying the pathogenesis of BPD.

## Introduction

Borderline personality disorder (BPD) is a mental disorder characterized by a pervasive pattern of instability in affect regulation, interpersonal relationships, impulse control and self-image [1]. Clinical signs include impulsive aggression, emotional dysregulation, repeated self-injury, and chronic suicidal tendencies, which make BPD patients frequent users of mental-health resources [2,3]. The nine criteria for borderline personality disorder are stated by the Diagnostic and Statistical Manual of Mental Disorders (DSM-IV) [4]. The lifetime prevalence of BPD has been estimated to be about 6% [5]. Additionally, findings showed that BPD was more common in women than in men (about 70% and 30%, respectively) [6]. The disorder often co-occurs with mood, anxiety and substance abuse disorders, and is also associated with other personality disorders. The disorder is characterized by severe psychosocial impairment [7] and a high mortality rate due to suicide. Up to 10% of patients commit suicide, a rate almost 50 times higher than in the general population. Several measures (semi-structural interviews) are highly reliable in the care of these patients [8-10]. Causal factors for BPD are only partly known, but genetic factors [11-13] and adverse events during childhood, such as physical and sexual abuse [14], contribute to the development of the disorder. Current strategies are focusing on the neurobiological underpinnings of the disorder, functional imaging [15], identifying endophenotypes within this heterogeneous diagnostic category, and the development and dissemination of better and more cost-effective disorder-specific psychotherapeutically treatments and pharmacological interventions [16].

Aberrant epigenetic modifications are associated with altered gene expression and this may contribute to several illnesses including cancer and psychiatric diseases (e.g. major depressions and schizophrenia) [9,17-22]. Epigenetic inactivation of disease related genes is accomplished by increased cytosine methylation at CpG sites and decreased acetylations of histones. Interestingly, several psychiatric diseases (e.g. schizophrenia, major depression, migraine, epilepsy, bipolar disorder) are therapeutically treated with valproic acid (VPA). Functionally VPA inhibits histone deacetylases and may increase expression of disease-related genes. 

For borderline personality disorder the epigenetic regulation of disease-associated genes has not been investigated in detail. In a first study, we have analyzed the DNA methylation pattern of 14 neuropsychiatric genes and found significantly elevated methylation levels of *5-hydroxytryptamine* (*serotonin*) receptor *2A*, *glucocorticoid receptor* gene (*NR3C1*), *monoamine oxidase A* and *B* and *soluble COMT* for BPD patients [9]. Others have confirmed this increased methylation frequency for *NR3C1* and brain-derived neurotrophic factor (BDNF) in BPD [23-25] This data suggests that aberrant epigenetic regulation of neuropsychiatric genes may contribute to pathogenesis of BPD [9]. 

The aim of this study was to identify novel target genes that may exhibit aberrant DNA methylation frequencies in BPD patients. Therefore we performed genome wide methylation profiling utilizing Illumina's bead chip technology. Significant changes were confirmed by quantitative pyrosequencing. Here we report increased methylation of *APBA2, APBA3, GATA4, KCNQ1, MCF2, NINJ2* and *TAAR5* at gene-specific CpG sites in female BPD blood samples compared to controls.

## Material and Methods

### BPD patients and controls

Whole blood samples for 24 female BPD patients and 11 female controls were obtained from the Psychiatric Hospital in Münsterlingen, Switzerland. All patients signed informed consent at initial clinical investigation. The study was approved by the local ethic committees (Kantonale Ethikkommission Thurgau and Ethik-Kommission am Fachbereich Medizin Justus-Liebig Universität Gießen). Diagnosis of BPD was established by an experienced psychiatrist (Dr. G. W. Dammann). Clinicopathological parameter of the female patients and controls are summarized in Table 1 and Table S1. Genomic DNA from whole blood was isolated by Nucleo Spin L Blood (Macherey and Nagel, Düren, Germany).

**Table 1:** Summarized data of the analyzed BPD patients and control persons.

	**BPD patients^*a*^ (n=24)**	**control persons (n=11)**
female	24	11
mean age	33 ± 11	32 ± 7
criteria 1	75% (18/24)	-
criteria 2	54% (13/24)	-
criteria 3	92% (22/24)	-
criteria 4	63% (15/24)	-
criteria 5	88% (21/24)	-
criteria 6	88% (21/24)	-
criteria 7	58% (14/24)	-
criteria 8	63% (15/24)	-
criteria 9	54% (13/24)	-
observed positive diagnosis	100% (24/24)	-
acute self injuring behavior	63% (15/24)	-
prior self injuring behavior	88% (21/24)	-
suicide background	83% (20/24)	-
nicotine consumption	71% (17/24)	36% (4/11)
alcohol abuse	25% (6/24)	-
additional drug abuse	17% (4/24)	-
traumatic experience in the past	63% (15/24)	-

^a^ more details are listed in Table S1.

### Infinium HumanMethylation27 BeadChip

For the bead chip array 500 ng of genomic DNA was treated with bisulfite [9] and Infinium bead chip analysis was performed by Life & Brain GmbH (Bonn, Germany). The HumanMethylation27 panel targets CpG sites located within the proximal promoter regions of transcription start sites of 14,475 consensus coding sequences (CCDS) in the NCBI Database (Genome Build 36). This bead chip technology allows researchers to interrogate 27,578 highly informative CpG sites per sample at single-nucleotide resolution. Arrays were hybridized and scanned at Life & Brain GmbH according to the manufacturer's instructions. Relative methylation levels and differential methylation were calculated with default settings in GenomeStudio software (Illumina, San Diego, USA). All subsequent calculations were based on average beta values and DiffScores as extracted from the GenomeStudio analysis. Bead chip methylation data has been deposited at the Gene Expression Omnibus (GEO) repository (GSE52222).

### Methylation analysis

DNA methylation of *APBA2, APBA3, GATA4, HLCS, KCNQ1, MCF2, NINJ2* and *TAAR5* was determined by bisulfite pyrosequencing [26]. Bisulfite treatment of genomic DNA was done as described previously [9]. For pyrosequencing 100 ng of bisulfite treated DNA was amplified in a reaction buffer containing 0.2 mM dNTP mix, 1.5 mM MgCl_2_ and 1.5 U Taq polymerase (InViTek GmBH, Berlin, Germany) for 40 cycles with 10 pmol each of forward and biotinylated reverse primer and sequenced with an internal primer (table S2). Pyrosequencing was performed in PyroMark Q24 according to the PyroMark Gold Q24 Reagents Handbook (Qiagen, Hilden, Germany). Pyrosequencing was done for two to three independent bisulfite reactions and average methylation frequency for each CpG site was calculated.

### Statistical evaluation

Statistical analysis was performed with the two-tailed t-test using Excel Version 14.3.1 (Microsoft Cooperation, Redmond, WA, USA). All reported p-values are considered significant for p≤0.05.

## Results

### DNA methylation in BPD patients by 27K bead chip technology

**Figure 1:**
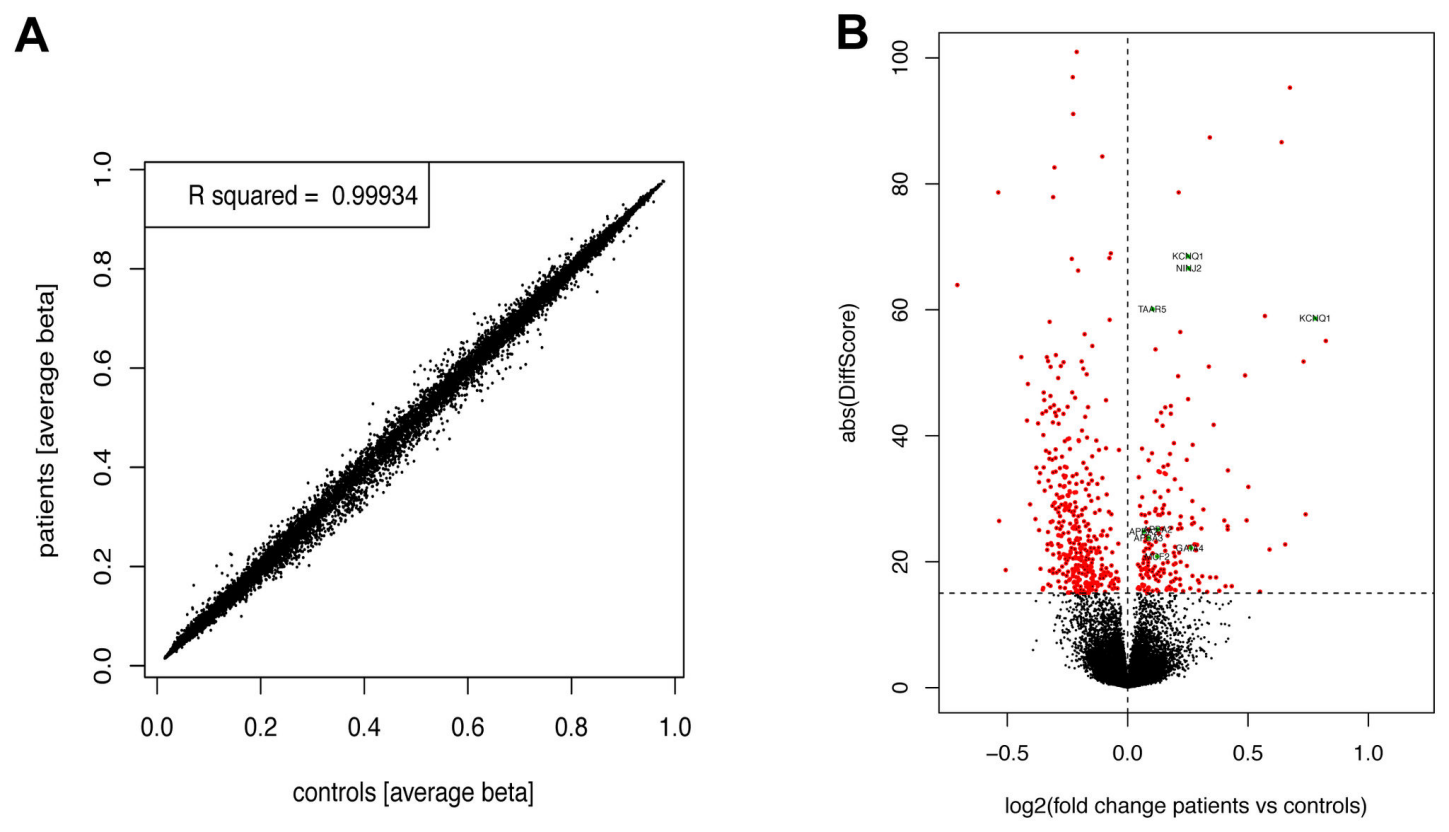
Methylation rate at 27,578 individual CpG target sites from 24 female BPD patients and 11 female control samples. Scatter plot of pairwise comparison between female BPD patients and controls (A) and volcano plot for visualization of differentially methylated probes between both groups (B). Methylation levels of 27,578 CpGs sites were revealed by HumanMethylation27 bead chip technology and the average beta-value (beta) and the absolute DiffScore for patients and control groups was determined. Beta is the ratio of the methylated signal to the sum of unmethylated and methylated signal. The DiffScore is a transformation of the p-value that provides directionality to the p-value based on the difference between the average signal in the reference group vs. the comparison group. The formula is: DiffScore = 10*sgn(µcond-µref)*log10p; For a p-value of 0.05 the DiffScore is ~13. 259 CpGs exhibited a significantly higher DiffScore (≥13). Analyzed CpGs of *APBA2, APBA3, GATA4, HLCS, KCNQ1, MCF2, NINJ2* and *TAAR5* are indicated.

 In our previous study we have reported that an increased methylation of CpG sites associated with neuropsychiatric genes occurs in the blood samples of borderline personality disorder (BPD) patients [9]. To reveal novel target sites that exhibit differential DNA methylation levels in the blood of BPD patients, we performed genome wide methylation analysis utilizing the HumanMethylation27 bead chip technology [27]. The methylation rate at 27,578 individual CpG target sites from 24 female BPD patients and 11 female control samples were analyzed on these bead chips. Data were analyzed by scatter plot and volcano plot for visualization of differentially methylated CpG sites (Figure 1). The scatter plot shows a high correlation between methylation levels of female BPD patients and controls (R^2^=0.99934, Figure 1A). However 259 CpG sites exhibited a significant different methylation level in patients (Figure 1B). For further verifications, we have selected several CpGs that show significant higher methylation in patients compared to controls (Figure 1B and Tab. 2). For *holocarboxylase synthetase* (*HLCS*) and *potassium voltage-gated channel KQT-like subfamily* member *1* (*KCNQ1*) such an increased methylation (1.75 and 1.72, respectively) was found (Tab. 2). Additionally we found several CpG sites that exhibited only slight but significant increase in DNA methylation (Tab. 2). For the *amyloid beta* (*A4*) *precursor protein-binding family A member 2* (*APBA2*) and member *3* (*APBA3*) a 1.05 to 1.1 fold increase and for GATA4 a 1.2 fold increase in CpG methylation was detected. Similar result were observed for CpG sites of the *MCF2 cell line derived transforming sequence* (*MCF2*): 1.09, *ninjurin 2* (*NINJ2*): 1.19 and *trace amine associated* receptor *5* (*TAAR5*): 1.07 (Tab. 2).

**Table 2:** Differentially methylated CpGs by 27K bead chip and pyrosequencing.

**Gene (Target ID)**	**27K bead chip**		**pyrosequencing**	
	Fold change**^*a*^**	T-test (p-value)	Fold change**^*a*^**	T-test (p-value)
APBA2 (cg21917349)	1.09	0.007	1.07	0.05
APBA2 (cg12044210)	1.05	0.014	1.08	0.08
APBA3 (cg20366831)	1.06	0.031	1.08	0.02
GATA4 (cg17795240)	1.20	0.012	1.10	0.47
HLCS (cg27519424)	1.75	0.002	1.02	0.38
KCNQ1 (cg17820828)	1.19	0.098	1.60	0.001
KCNQ1 (cg19728223)	1.72	0.008	n.a.	n.a.
MCF2 (cg21557231)	1.09	0.025	1.10	0.02
NINJ2 (cg20781967)	1.19	0.0007	1.17	0.001
TAAR5 (cg17829936)	1.07	0.057	1.05	0.13

^a^ methylation of patients versus controls, n.a. not analyzed

### Increased methylation of gene associated CpG sites in BPD patients

**Figure 2:**
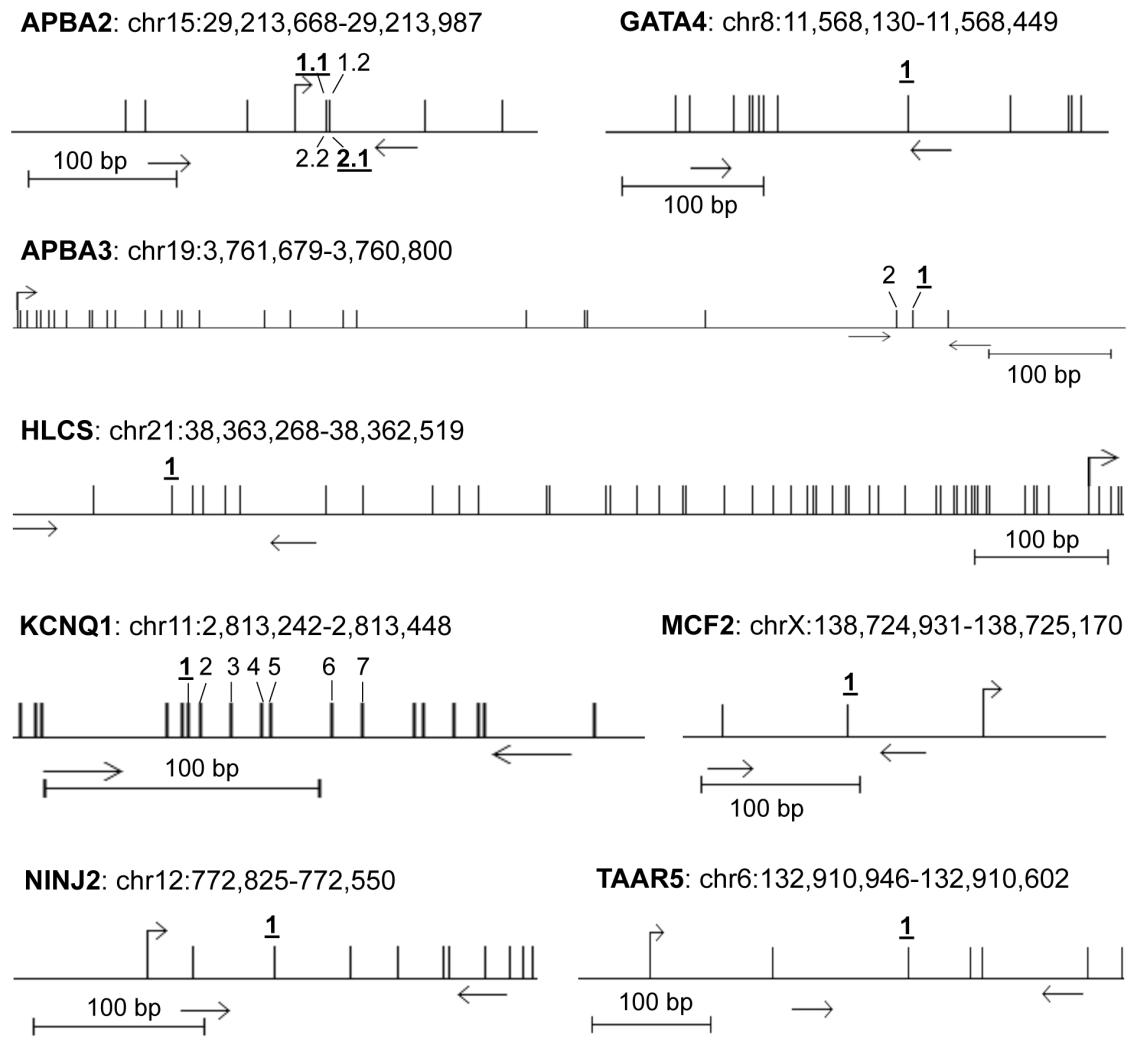
Map of the investigated genomic regions. Positions of the analyzed CpG sites of *APBA2, APBA3, GATA4, HLCS, KCNQ1, MCF2, NINJ2* and *TAAR5* are shown (http://genome.ucsc.edu). CpGs are indicated with vertical lines and the analyzed CpGs are numbered. The bold and underlined CpG sites were identified as differentially methylated in the 27K bead chip assay. Primers and transcriptions start sites are marked with arrows. Graphics were generated with the python.vs.cobra program (https://launchpad.net/python.vs.cobra).

**Figure 3:**
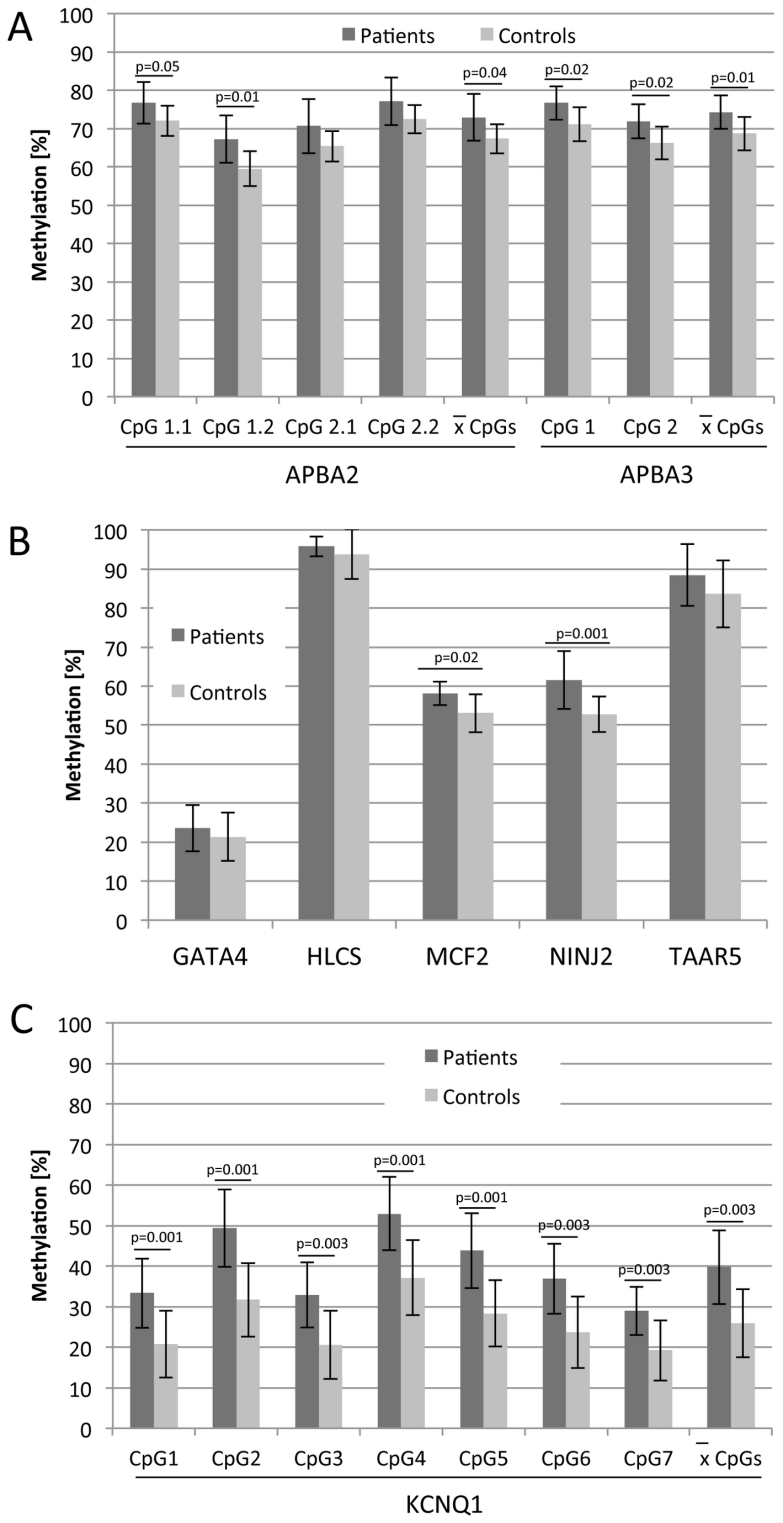
Methylation analysis of *APBA2, APBA3, GATA4, HLCS, KCNQ1, MCF2, NINJ2* and *TAAR5* by bisulfite pyrosequencing. PCR products obtained from three independent bisulfite treated DNA of female BPD patients (n=9) and controls (n=8) were analyzed. Average methylation levels ± SD of individual CpG sites from three independent pyrosequencing reactions are indicated and summarized for all CpGs. Significant p-values are indicated (two-tailed t-test).

**Figure 4:**
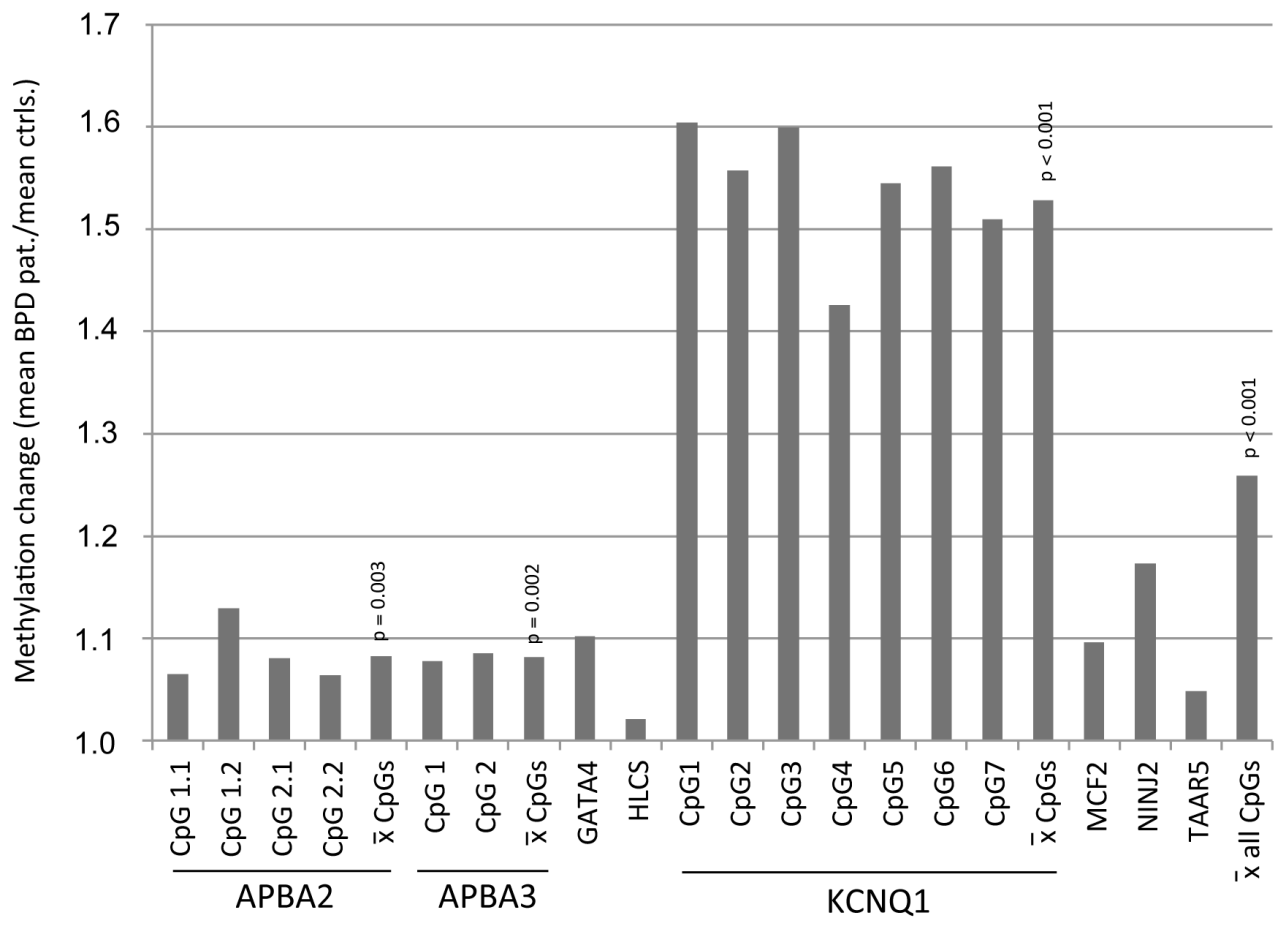
Methylation changes between female BPD patients and controls. The quotient between mean methylation levels at individual CpG sites and combined sites for *APBA2, APBA3, GATA4, HLCS, KCNQ1, MCF2, NINJ2* and *TAAR5* in BPD patients and controls were calculated and plotted. P-values are indicated (two-tailed t-test).

To validate our bead chip results, we performed quantitative pyrosequencing for *APBA2, APBA3, GATA4, HLCS, KCNQ1, MCF2, NINJ2* and *TAAR5* (Figure 2 and Figure 3). Most identified target CpG sites were located at non-CpG island regions and are depicted in Figure 2. For *APBA2* two target CpG sites and two additional CpGs were analyzed from which two were located at the upper strand (target CpG1.1 and CpG1.2) and two on the complementary lower strand (target CpG2.1 and CpG2.2). For controls the average methylation frequencies were 72% for CpG1.1, 59% for CpG1.2, 65% for CpG2.1 and 72% for CpG2.2 (Figure 3A). In BPD patients increased frequencies were observed: 77%, 67%, 71% and 77%, respectively (Figure 3A). Thus in average a significant higher methylation of 5.6% was observed in BPD (72.9%) compared to controls (67.3%) and a 1.08 fold higher methylation was found (p<0.05; Figures 3A and 4). Similar results were also found for the two CpG sites (target CpG1 and CpG2) analyzed in the coding region of *APBA3* (Figure 2 and Figure 3A). In controls CpG1 showed 71% and CpG2 66% methylation and a significant 5.6% increase was found for BPD patients (77% and 72%, respectively). In average we observed a 1.08 fold increase in methylation of *APBA3* in BPD patients (74.3%) compared to controls (68.7%; Figures 3A and 4). 

For *GATA4* methylation was higher in BPD patients (24%) compared to controls (21%), however this increase (1.1) was not significant (Figures 3B and 4). In the bead chip analysis the highest methylation change (1.75 fold increase in BPD) was found for *HLCS* (Tab. 2). However, our pyrosequencing results show only minor changes at the target CpG site (Figure 3B). In BPD patients 96% and in controls 94% methylation and this 2% increase (1.02 fold change) in BPD was not significant (Figures 3B and 4). The methylation of the target CpG of *MCF2* was higher in BPD patients (58%) compared to controls (53%) and this 1.1 fold increase was significant (p=0.02; Figures 3B and 4). Similar results were also found for *NINJ2* (Figures 2 and 3). The target CpG of *NINJ2* showed significantly higher (1.17 fold; p<0.001) methylation in BPD patients (62%) compared to controls (53%; Figures 3B and 4). For *TAAR5* an increased methylation was observed in BPD patients (88%) compared to controls (84%), however this 1.05 fold increase was insignificant (Figures 3B and 4). 

Next we analyzed seven CpGs in the coding region of *KCNQ1*. For controls the average methylation levels were 21% for CpG1, 32% for CpG2, 21% for CpG3, 37% for CpG4, 28% for CpG5, 24% for CpG6 and 19% for CpG7 (Figure 3C). In BPD patients significant higher levels were observed at all CpGs: 33%, 49%, 33%, 53%, 44%, 37% and 29%, respectively (Figure 3C). Thus, in average a significant 14% higher methylation was observed in BPD (40%) compared to controls (26%) and a 1.54 fold higher rate was calculated (p<0.001; Figures 3C and 4).

In figure 4 the fold increase of methylation levels in BPD patients compared to controls is plotted. Our data indicate increased methylation frequencies at all 18 analyzed CpGs and the average methylation level was significantly 1.26 fold higher in BPD patients (p<0.001). Aberrant methylation was not significantly associated with a specific individual or with a personality trait (e.g. traumatic experience or drug abuse). Interestingly our pyrosequencing data showed with exception of *HLCS*, a similar increase in DNA methylation compared to the bead chip analysis for most of the analyzed CpGs (Figure 4 and Tab. 2). 

To analyze if aberrant methylation of target CpG sites correlates with altered gene expression, we utilized human cells, which were treated with 5-aza-2’-deoxycytidine, a potent inhibitor of DNA methyltransferases [28]. After four days of treatment methylation levels at two CpG sites (CpG1.1 and CpG2.1) of *APBA2* and CpG1 of *APBA3* were investigated by bead chip assay and gene expression of *APBA2* and *APBA3* was analyzed by quantitative RTPCR (Figure S1). Methylation analysis revealed a decreased methylation of *APBA2* (15%; 0.84 fold) and *APBA3* (3%; 0.97 fold) compared to untreated control cells (Figure S1A). In parallel expression of *APBA2* and *APBA3* was increased by 1.25 and 1.12 fold, respectively (Figure S1B). Similar results were also observed for other genes (e.g. *NINJ2*; data not shown). These data suggest that small variations in DNA methylation correlate with relevant changes in expression of *APBA2* and *APBA3*. In summary our results indicate an increase in DNA methylation in samples of BPD patients compared to controls at several gene specific CpG sites and this may lead to a decreased expression of the respective genes.

## Discussion

 In our study we aimed to reveal novel gene associated CpG sites that exhibit aberrant DNA methylation in borderline personality disorder (BPD). Thus we performed a genome wide methylation screen utilizing the HumanMethylation27 bead chip technology [27,29]. By this unbiased approach we identified several CpG sites, which exhibited significantly increased methylation in the blood of BPD patients compared to controls (Table 2). This increased methylation was confirmed by quantitative pyrosequencing for *APBA2* (1.08 fold)*, APBA3* (1.08 fold), *GATA4* (1.1 fold), *KCNQ1* (1.54 fold), *MCF2* (1.10 fold), *NINJ2* (1.17 fold) and *TAAR5* (1.05 fold) (Figure 4). Only for *HLCS* the 1.75 fold increased methylation was not confirmed by pyrosequencing (Tab. 2). In our previous work we have reported that increased methylation of *5-hydroxytryptamine* (*serotonin*) receptor *2A* (1.24 fold), *glucocorticoid receptor* gene (*NR3C1*) (1.22 fold), *monoamine oxidase A* (1.05 fold), *monoamine oxidase B* (1.08 fold) and *soluble COMT* (1.07 fold) occurs in BPD [9]. Thus our data show a similar increased methylation rate in the blood of BPD patients as observed previously for certain genomic regions.

 To confirm that this difference in methylation is of biological significance we have performed expression and methylation analysis of *APBA2* and *APBA3* in human cells (Figure S1). We revealed that a decrease of 0.84 and 0.97 fold in methylation levels correlated with an increase of 1.25 and 1.12 fold in mRNA levels of *APBA2* and *APBA3*, respectively. This data suggests that small differences in DNA methylation result in differential expression of the respective genes. Others have also observed a relationship between increased methylation of regulatory regions of rDNA and reduced rRNA expression in the hippocampus of suicide subjects [30]. For ethical reasons brain samples were not available in our study and therefore we utilized blood samples of female BPD patients and controls. 

 APBA2 and APBA3 are both neuronal adaptor proteins, which are also termed MINT2/X11-Beta and MINT3/X11L2 (MINT; methylated in tumors). Aberrant methylation of *APBA2/MINT2* and *APBA3/MINT3* were not previously reported in psychiatric disease however frequently observed in cancer [31]. Thus these genes may represent sites that are frequently targeted by DNA methyltransferases for aberrant DNA methylation. Functional over-expression of APBA1/X11-Beta has been shown to decrease the production of amyloid-β, a toxic peptide deposited in Alzheimer's disease brains [32]. APBA/X11 interaction with β-Amyloid precursor protein (APP) modulates its cellular stabilization and reduces amyloid β-protein secretion [33]. *APBA2* and *APBA3* encode transactivators that regulate the APP signaling network [32,34]. Hypermethylation of CpG sites at the *APBA2* promoter suppressed its activity [35]. Our expression analyses also suggest that an increased methylation of the analyzed CpGs correlates with a reduced expression of *APBA2* and *APBA3* (Figure S1). Thus it is tempting to speculate that epigenetic alteration of *APBA2* and *APBA3* may contribute to borderline personality disorder. 

 Additionally we identified a CpG site of *NINJ2* that exhibit 1.2 fold hypermethylation in BPD samples. NINJ2 is a novel homophilic adhesion molecule, which is expressed in mature sensory and enteric neurons [36]. NINJ2 promotes neurite outgrowth from primary cultured dorsal root ganglion neurons, presumably via homophilic cellular interactions [36]. It is interesting to note that genetic variants of *NINJ2* have been associated with a decreased risk of Alzheimer’s disease [37]. A recent study has shown that common psychosocial stressors in women related to longstanding distress and increased risk of Alzheimer's disease [38]. However a correlation between BPD and Alzheimer’s disease has not been reported yet.

Trace amine-associated receptors (TAAR) are a distinct subfamily within the family of G protein-coupled receptors and consist of nine TAAR members in mammals [39]. *TAAR* genes are selectively expressed in the olfactory epithelium [40]. Mouse Taar3, Taar4 and Taar5 recognize volatile amines found in urine: Taar4 detects a compound linked to stress, whereas Taar3 and Taar5 detect compounds like pheromones that are enriched in male urine [40]. Rat Taar1 is stimulated by trace amines and psychoactive substances like MDMA or LSD and activates adenylyl cyclase signaling pathway [41]. For BPD patients substance abuses are commonly observed and deregulated TAARs may alter cellular response to these compounds. In our screen *TAAR1* was not identified as an aberrantly methylated gene. However since only one probe for *TAAR1* is present on our bead chip this site may not be representative for the whole *TAAR1* gene.

The MCF2/DBL encodes an oncogenic guanine nucleotide exchange factor that exerts control over some members of the Rho family of small GTPases. Expression of *Mcf2/Dbl* is mainly found in the brain and the gonads [42]. *Mcf2*/*Dbl* knockout mice reportedly display shorter dendrites in distinct populations of *Dbl*-null cortical pyramidal neurons, which suggest a function for Mcf2/Dbl in dendrite elongation [42,43]. Thus increase methylation of *MCF2* in BPD may have an impact on growth of dendrites. It will be interesting to analyze methylation of *MCF2* in the brain of suicide victims, however due to ethical issues this was not feasible in our study.

For *GATA4* we found an increase in methylation in BPD patients, however this increase was not significant for the pyrosequencing. GATA4 encodes a member of the GATA family of zinc-finger transcription factors that recognize the GATA sequence, a motif present in the promoters of many genes. A significant association between GATA4 and alcohol dependence at the gene level was demonstrated [44]. GATA4 is thought to regulate genes involved mainly in embryogenesis and in myocardial differentiation and function [45]. Further studies are needed to identify potential causal functional role of GATA4 contributing to BPD.

The *KCNQ1* gene encodes a voltage-gated potassium channel, which is required for repolarization phase of the cardiac action potential. Mutations of *KCNQ1* are associated with hereditary long QT syndrome 1 and other syndromes [46]. The genomic region of *KCNQ1* exhibits tissue-specific imprinting [47] and in whole blood certain CpGs show 40 to 60% methylation [48]. We observed 25 to 40% methylation, but we have analyzed a different region, which was identified by the bead chip technology. Our result indicates a 1.5 fold increased methylation in blood samples of female BPD patients (Figures 3C and 4). It is interesting to note that we have identified two distinct CpGs by our screen that exhibit hypermethylation (Table 2). Hypermethylation of *KCNQ1* occurs also in the sperm DNA of infertile males [49]. Loss of imprinting has been reported in cancer [50]. Since in our study we have only analyzed samples of women, gender specific variations in DNA methylation due to genomic imprinting may not be relevant for this difference. It will be interesting to dissect the role of aberrant methylation of *KCNQ1* in BPD in further details.

 We selected *HLCS* for further analysis since in the bead chip analysis high hypermethylation (1.8 fold) in BPD patients was found (Table 2). However in our pyrosequencing verification this significant increase in methylation could not be confirmed (Figure 3). For flanking CpG sites a slight decrease in methylation was observed in BPD (data not shown). Thus increased methylation of *HLCS* in bead chip was not verified by subsequent analysis and *HLCS* may not represent a target gene that exhibits epigenetic alterations in BPD.

Causal factors for borderline personality disorder are only partly revealed, however genetic factors [11-13] and adverse events during childhood, such as sexual and physical abuse [14], contribute to the pathogenesis of BPD. Other reports have suggested a role for environmentally mediated aversive events in the development of BPD and have found an association between psychotraumatization during childhood and the diagnosis of BPD [51-56]. In a family environment, early maltreatment (neglect and childhood abuse) may be particularly involved in producing long-term epigenetic changes [57]. In mammalian models, postnatal maternal care has been linked to epigenetic alterations by aberrant DNA methylation [58,59] and early adversity was shown to alter the offspring epigenome at the glucocorticoid receptor gene (*NR3C1*) promoter [60]. Previously we have observed increased methylation of *NR3C1* in BPD patients [9]. Due to the low number of patient samples analyzed in our study, aberrant methylation of the gene specific CpG sites was not significantly associated with any clinicopathological parameter. It will be interesting to analyze a larger cohort of patients and to correlate DNA methylation pattern with early adversity (e.g. maltreatment or child hood abuse), other traumatic experiences or alcohol and drug abuse. In future research it will be also important to investigate in detail if variations in blood cells, medications, physical activity and other conditions (e.g. inflammation, menstrual cycle and contraception) contribute to the observed differences in DNA methylation in blood cells, since these parameters may impact the blood cell population and cell-type specific DNA methylation levels [61,62]. Due to ethical issues the analysis of brain tissue (e.g. hippocampus) was not feasible in our study. It has been suggested that in brain and blood the methylation pattern of catechol-*O*-methyltransferase and other genes are nearly identical [63,64]. Tissue specific methylation of certain developmental genes has been reported [65,66]. Thus the methylation pattern of blood and brain tissues could be also divergent [65]. It will be important to compare DNA methylation levels in brains of BPD patients and controls. 

Here we report that increased methylation of certain gene associated CpG sites significantly occurs in BPD patients. It is interestingly to note that three (*APBA2*, *APBA3* and *NINJ2*) of these genes are correlated with Alzheimer’s disease. However progression of BPD to dementia has not been investigated or reported yet. Our results suggest that epigenetic deregulation of several gene associated CpG sites may be attributed to the pathogenesis of BPD.

## Supporting Information

Figure S1 **Expression and demethylation of APBA2 and APBA3.** A. Methylation analysis of APBA2 (cg21917349; CpG 1.1 and cg12044210 ; CpG 2.1) and APBA3 (cg20366831) is shown for the lung cancer cell line A549 after 4 days of aza (5-aza-2’-deoxycytidine) treatment (0 and 5 µM). Bisulfite treated DNA was analyzed by Illumina bead chip technology. B. Expression analysis of APBA2 and APBA3 is shown after aza treatment. RNA isolated from A549 cells was analyzed by quantitative RTPCR with primers APBA2RTF1 5’-CCACCTGCCAAGGCATCATCAAG, APBA2RTR1 5’-GCTCAGCAATGCCCCCTCTCATG APBA3RTF1 5’-TGCTCACCGAGGCCTATGGCG, APBA3RTR1, 5’-CCATGGAGGCGAAGGCACAGTG and normalized to GAPDH expression.
(TIF)

Table S1 **Clincopathological parameter of BPD patients.**
(DOCX)

Table S2 **Primers for methylation analysis.**
(DOCX)
