# Supplementary figures and images for: Aberrant Methylation of Gene Associated CpG Sites Occurs in Borderline Personality Disorder
Source: PLoS One. 2013 Dec 19;8(12):e84180. doi: 10.1371/journal.pone.0084180 (PMC3868545; doi:10.1371/journal.pone.0084180)

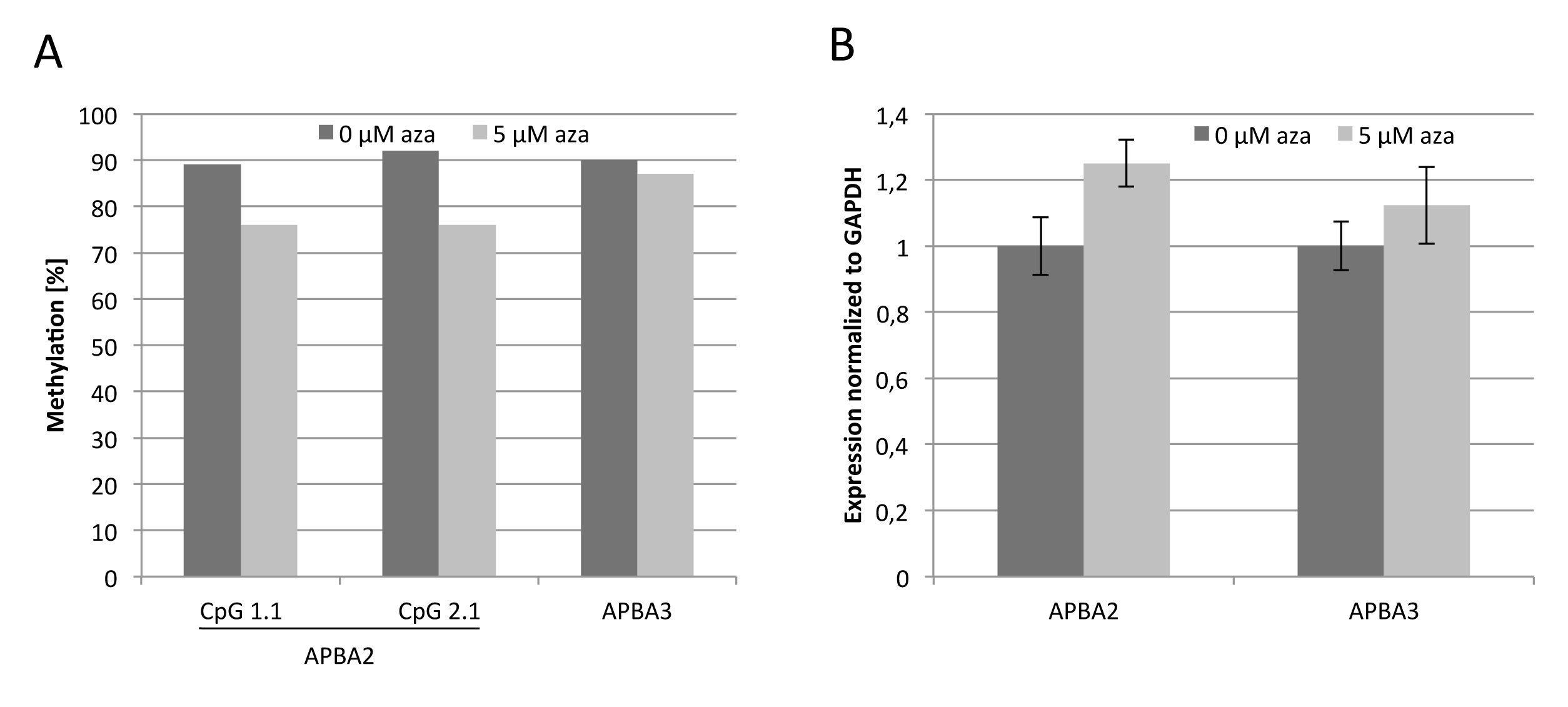

Supplement: Figure S1 — Expression and demethylation of APBA2 and APBA3. A. Methylation analysis of APBA2 (cg21917349; CpG 1.1 and cg12044210 ; CpG 2.1) and APBA3 (cg20366831) is shown for the lung cancer cell line A549 after 4 days of aza (5-aza-2’-deoxycytidine) treatment (0 and 5 µM). Bisulfite treated DNA was analyzed by Illumina bead chip technology. B. Expression analysis of APBA2 and APBA3 is shown after aza treatment. RNA isolated from A549 cells was analyzed by quantitative RTPCR with primers APBA2RTF1 5’-CCACCTGCCAAGGCATCATCAAG, APBA2RTR1 5’-GCTCAGCAATGCCCCCTCTCATG APBA3RTF1 5’-TGCTCACCGAGGCCTATGGCG, APBA3RTR1, 5’-CCATGGAGGCGAAGGCACAGTG and normalized to GAPDH expression. (TIF) [file pone.0084180.s001.tif]
