# Supplementary material for: Aberrant Methylation of Gene Associated CpG Sites Occurs in Borderline Personality Disorder
Source: PLoS One. 2013 Dec 19;8(12):e84180. doi: 10.1371/journal.pone.0084180 (PMC3868545; doi:10.1371/journal.pone.0084180)
Supplement: Table S1 — Clincopathological parameter of BPD patients. (DOCX) [file pone.0084180.s002.docx]

| patient | gender | age | crit. 1 | crit. 2 | crit. 3 | crit. 4 | crit. 5 | crit. 6 | crit. 7 | crit. 8 | crit. 9 | positive diagnosis | acute self injuring behavior | prior self injuring  behavior | suicide background | nicotine consumption | alcohol abuse | additional drug abuse | prior  traumatic experience | co-diagnosis |
| --- | --- | --- | --- | --- | --- | --- | --- | --- | --- | --- | --- | --- | --- | --- | --- | --- | --- | --- | --- | --- |
| 1 | f | 40 | + | - | + | + | + | + | + | - | - | **+** | + | + | - | - | - | - | + | - |
| 2 | f | 33 | + | - | + | + | + | + | + | + | + | **+** | + | + | + | + | - | - | - | - |
| 3 | f | 24 | + | + | + | + | + | + | + | + | + | **+** | - | + | + | + | - | - | + | - |
| 4 | f | 40 | + | - | + | + | - | + | + | + | + | **+** | - | + | + | + | - | - | - | F10 |
| 5 | f | 52 | + | - | + | + | + | - | + | - | + | **+** | - | - | + | - | - | - | + | - |
| 6 | f | 17 | + | + | + | + | + | + | + | + | + | **+** | + | + | + | + | - | - | + | - |
| 7 | f | 28 | + | - | + | + | + | + | - | + | + | **+** | + | + | + | - | - | - | + | F42.2 |
| 8 | f | 47 | + | - | + | - | + | + | + | - | - | **+** | - | + | + | - | - | - | - | depression; anorexia |
| 9 | f | 51 | + | - | + | - | + | + | + | - | - | **+** | - | + | + | + |  | - | - | alcoholism |
| 11 | f | 33 | + | - | + | + | + | + | - | + | + | **+** | + | + | + | + | + | canabis | + | [narcissistic](http://dict.leo.org/ende?lp=ende&p=Ci4HO3kMAA&search=narcissistic&trestr=0x404) personality disorder |
| 12 | f | 18 | - | + | + | + | + | + | - | - | - | **+** | + | + | + | + | - | - | + | - |
| 13 | f | 26 | + | + | + | + | + | + | - | + | - | **+** | + | + | + | + | + | amphetamine | + | F19; ADHS, Polytox |
| 14 | f | 24 | + | + | + | - | + | - | + | - | + | **+** | + | + | + | - | - | - | - | pregnancy |
| 15 | f | 52 | + | - | - | + | + | + | + | + | - | **+** | - | - | + | + | + | temesta | + | F60.30; F33.1; F10.21; Z56/Z59/Z63 |
| 16 | f | 23 | + | + | + | - | + | + | - | - | + | **+** | + | + | + | + | - | - | + | - |
| 17 | f | 24 | + | + | + | - | + | + | + | - | - | **+** | + | + | + | + | - | - | + | - |
| 18 | f | 26 | - | + | + | - | + | + | + | + | - | **+** | + | + | + | + | - | - | + | - |
| 19 | f | 45 | + | + | + | - | - | + | - | + | + | **+** | - | - | - | - | - | - | - | F61 |
| 21 | f | 22 | - | + | + | + | + | + | - | - | - | **+** | + | + | + | + | + | - | - | - |
| 22 | f | 36 | + | + | + | - | + | + | - | + | + | **+** | + | + | + | - | - | - | - | - |
| 23 | f | 22 | + | + | - | + | - | + | + | + | - | **+** | + | + | - | + | + | + | + | F33.1 |
| 24 | f | 49 | - | - | + | + | + | + | + | + | + | **+** | - | + | + | + | - | - | - | - |
| 25 | f | 38 | - | + | + | + | + | + | - | + | + | **+** | - | + | + | + | + | - | - | - |
| 26 | f | 20 | + | - | + | - | + | + | - | + | - | **+** | + | + | - | + | - | - | + | - |

**Table S1.** Clincopathological parameter of BPD patients.

DSM IV: A pervasive pattern of instability of interpersonal relationships, self-image, and affects, and marked impulsivity beginning by early adulthood and present in a variety of contexts, as indicated by five (or more) of the following:

crit. 1: frantic efforts to avoid real or imagined abandonment. Note: Do not include suicidal or self-mutilating behavior covered in crit. 5.

crit. 2: a pattern of unstable and intense interpersonal relationships characterized by alternating between extremes of idealization and devaluation

crit. 3: identity disturbance: markedly and persistently unstable self image or sense of self

crit. 4: impulsivity in at least two areas that are potentially self-damaging (e.g., spending, sex, substance abuse, reckless driving, binge eating). Note: Do not include suicidal or self-mutilating behavior covered in crit. 5.

crit. 5: recurrent suicidal behavior, gestures, or threats, or self-mutilating behavior

crit. 6: affective instability due to a marked reactivity of mood (e.g., intense episodic dysphoria, irritability, or anxiety usually lasting a few hours and only rarely more than a few days)

crit. 7: chronic feelings of emptiness

crit. 8: inappropriate, intense anger or difficulty controlling anger (e.g., frequent displays of temper, constant anger, recurrent physical fights

crit. 9: transient, stress-related paranoid ideation or severe dissociative symptoms

F10: mental and behavioural disorder due to alcoholism

F42.2: compulsion minds and –acts, mixed

F19: psychic and behavioural disorder due to multiple substance abuse and consumption of additional psychotropic substances

F60.30: emotionally instable personality disorder: impulsive type

F33.1: recurrent depressive disorder, actual moderately episode

F10.21: psychic and behavioural disorder due to psychotropic substances; dependence syndrome

Z56: problems related to employment and unemployment

Z59: problems related to housing and economic circumstances

Z63: other problems related to primary support group, including family circumstances.
