# Supplementary material for: Aberrant Methylation of Gene Associated CpG Sites Occurs in Borderline Personality Disorder
Source: PLoS One. 2013 Dec 19;8(12):e84180. doi: 10.1371/journal.pone.0084180 (PMC3868545; doi:10.1371/journal.pone.0084180)
Supplement: Table S2 — Primers for methylation analysis. (DOCX) [file pone.0084180.s003.docx]

Table S2. Primers for methylation analysis

| **primer** | **sequence** | **product size (bp)** |
| --- | --- | --- |
| APBA2BSU1 | 5´-TTGTTATTTATATTTGTTTTTTTGTGGGTG | 168 |
| APBA2BSL1 | 5´-TCTATCCACAAAACAATCCTAACCTATTAA |  |
| APBA2BSSeq1 | 5’- GTTTTTTTTTTTTATAGTTTTTTGAATATTT | sequencing primer |
| APBA2BSU2 | 5’-GTTTGTTTATAGGATAGTTTTGGTTTATTG | 161 |
| APBA2BSL2 | 5’-CACACCTACCCCTCTATAAATAAATAAATAA |  |
| APBA2BSSeq2 | 5’-TTGGTGGTTTGTTTATAGGAGATTTAGTA | sequencing primer |
| APBA3BSU1 | 5´-ATTTTAGTTTGGGTGATAGAGTGAGGTTT | semi nested  105 |
| APBA3BSU11 | 5´-GAGGTTTTGTTTTAAAAAAAATAAATAAATT |  |
| APBA3BSL1 | 5´-CCTATATAAACAATACCCAACTAAAACCTAA |  |
| APBA3BSSeq | 5´-TTYGAAAAATAAAAAATTTGAGGTTT | sequencing primer |
| GATA4BSU2 | 5’-GGTTTTTTAAAGTGTTGGGATTATAGG | semi nested  171 |
| GATA4BSL1 | 5’-CACTACACTCCAACCTAATAACAAAACAAA |  |
| GATA4BSL2 | 5’-ACTAATCCAAAAAAAATTTACTTTATAACCC |  |
| GATA4BSSeq | 5’-TAATTAAATATTAAATTTTTTTTAGGATT | sequencing primer |
| HLCSBSU1 | 5’-GAATTTAGGGAGATATTTTATTGTTAGAA | 206 |
| HLCSBSL1 | 5’-CACTAATAACCAATAACATAATAAAACCTAA |  |
| HLCSBSSeq | 5’-GTTAGAAGATTATTGTAAAGAATATAAT | sequencing primer |
| KCNQ1BSU2 | 5'-GTTGGGTTGTTTGGGTTGGTAATT | 173 |
| KCNQ1BSL2 | 5'-AAACCTACAAAACCACCCCCTAAAAA |  |
| KCNQ1BSSeq | 5’-GGTTGGTAATTTGAGTTTTTTTTATT | sequencing primer |
| MCF2BSU1 | 5’-GGGTAGGAYGAGAGTAAAAAGTATGAGTT | 129 |
| MCF2BSL1 | 5’-CAACTCTCTTCCTAAAAACAAACTTAATAAA |  |
| MCF2BSSeq | 5’-TTTATAAAGATTTTTAGTATTTTATTTTAAA | sequencing primer |
| NINJ2BSU4 | 5’-TTTATAYGTGTGTGTAGGTGTATATTTTTTT | 172 |
| NINJ2BSL2 | 5'-AAACAAACTACRTAAACTCCTCCAAA |  |
| NINJ2BSSeq | 5’-GTGTGTGTAGGTGTATATTTTTTTTAGAGG | sequencing primer |
| TAAR5BSU2 | 5'-AGGTGAATGGGTTTTGTTTTAGGATAGT | 216 |
| TAAR5BSL2 | 5'-TAAAAAACAACACCAACAAACCCAAA |  |
| TAAR5BSSeq | 5’-TTTGGTTTGTGTAGTAGGTATGTTGATTA | sequencing primer |
